# Messrs. Bland and Comte on the Powers of Digitalis in Palpitatio Cordis

**Published:** 1826-07

**Authors:** 


### PALPITATIO CORDIS.

Irregular action in the central organ of the circulation is always an alarming phenomenon—especially to the young practitioner, and leads him often into false prognoses.

Dr. Blaud, Chief Physician to the Hospital of Beaucaire, has drawn the attention of his brethren recently to the subject of idiopathic palpitation, and relates some cases in elucidation. We shall first advert to the cases.

*Case* 1. A young woman, 21 years of age, in poverty and distress of mind, was carried to the hospital, on the 2d of April, 1825, having been two days ill. Excepting the functions of circulation and respiration, all the other organic actions appeared to be going on regularly. In the region of the heart were felt and distinctly heard the most violent and tumultuous palpitations, which sometimes subsided a little, but were readily excited by the slightest motion of the body. The pulse corresponded, being very quick, unequal, and intermitting. The respiration was embarrassed ; but the respiratory murmur could be distinctly heard in every part of the chest. The complexion was natural—no œdema of the lower extremities—no difficulty in lying in the horizontal position. The complaint had come on suddenly, without any evident cause, unless it was the mental chagrin under which she laboured. One grain of digitalis was prescribed thrice a-day. *Second day.* The same phenomena. Two grains of digitalis four times a-day. *Third day.* Some amelioration of the symptoms. Four grains of digitalis every three hours. In the evening the young woman ate largely of some salted and seasoned tongue, and was soon after seized with vomiting, lipothymia, and other alarming symptoms, which lasted all the night; but the pulse had become small, slow, and regular. *Fourth day.* No palpitation. From this time she continued well, and was soon discharged cured.

*Case* 2. A man, 48 years of age, entered the hospital on the 29th May, having laboured under palpitation of the heart for two months, which had come on suddenly, and without any known cause. The pulsations were rapid, tumultuous, and could not be analyzed by the stethoscope. The patient heard them distinctly himself. The pulse was small, quick, irregular, and intermitting. The respiratory murmur was heard throughout the chest, but yet there was oppression of the breathing. The lips were pale, and slightly blue. The man could not lie down at all—no swelling of the lower extremities—digestive organs regular in function. Two grains of digitalis thrice a-day. *30th* and *31st*. The same state—same prescription. *June* 1. Considerably better. The same medicine continued. *2d* and *3d*. The digitalis was increased. *4th*, The pulse was down to 38, but very irregular. The pulsation of the heart could scarcely be felt. Three grains of digitalis thrice a-day. The medicine was continued till the *8th*, and then suspended. The pulse was still at 38 in the minute, but regular, and all palpitation gone. The pulse soon arose to 42, but not higher till he left the hospital, which he did in a few days, feeling quite well.

*Case* 3. A woman 67 years of age, subject every winter to pulmonary catarrh, (her mother had been asthmatic) laboured under bronchitis for about a fortnight previous to the 25th December, 1824, when she was suddenly seized with great difficulty of breathing, and violent palpitation of the heart. These symptoms, more or less violent, continued till the first of June following, when the palpitation and dyspnœa became so constant and distressing, that she was obliged to apply for medical assistance. Antispasmodics of various kinds were given in abundance, but without any advantage. The paroxysms were exasperated in the night, and mitigated by day. The sulphate of quinine suspended them for three days and nights completely; but they then returned with the usual violence. She came under Dr. Blaud's notice on the 18th June, with the following symptoms, viz. pulsations of the heart strong, precipitate, tumultuous—pulse small, irregular, and intermittent—dyspnœa and orthopnœa considerable—respiratory murmur audible in all parts of the chest—cough frequent—expectoration mucous—no pain in the chest—lower extremities infiltrated. Digitalis was prescribed in pretty free doses, and in a few days the urine became plentiful, the œdema disappeared, and the palpitation and breathing were considerably relieved. The pulse came down to 48 in the minute, and afterwards to 40, the patient feeling very comfortable and able to lie down and enjoy sleep. In the midst of these favourable circumstances, she suddenly expired, with scarcely a moment's warning.

*Dissection*. Heart perfectly natural—lungs crepitous—abdominal viscera all sound—head and spinal marrow presenting no appearance of disease.

This case offers a striking example of what is termed, and perhaps properly so, *nervous palpitation*. Physiologists may say what they please about the heart being *independent* of the brain and spinal marrow—but few men have experienced any sudden emotion, without feeling in their own breasts, the *influence* of the mind in the central organ of the circulation. This observation is very old—at least as old as Erasistratus, who discovered the passion of Antiochus through this channel of information. But the heart is influenced by the brain and nervous system, where the passions or emotions of the mind are not at all concerned, and where we can see no connexion between the cause and the effect. The foregoing case is an example. There was no other cause but a nervous one, to disturb so long the function of the heart. We much doubt, however, whether this poor woman died of the disease for which she was under medical treatment. Such doses of digitalis as she was taking, with the action of the heart depressed to 40 pulsations in the minute, were sharp tools to work with! It is true, the œdematous swellings, the dyspnœa, and the palpitations were removed, or nearly so, by digitalis—and the patient was just on the point of being *cured*—when unfortunately, she expired. By an unlucky association of ideas, we are reminded of the Frenchman, who had just brought his horse to the point of living without food—when, malheureusement, the poor animal took it into his head to die.*

M. Blaud enters into some reflexions on the nature of this nervous affection, which gives rise to palpitation of the heart. He concludes that it is not *inflammatory*, since it generally gives way under the use of antispasmodics. Neither is it purely *adynamic*, since the inordinate action of the heart is increased by stimulants. To this last observation we cannot entirely subscribe, as we have seen palpitation of the heart subside on taking wine. Still the observation is, generally speaking, correct. The nature then of this nervous affection, Dr. B. thinks, is peculiar, or *sui generis*; and its treatment empirical. Be this as it may, he undertakes to prove that these nervous palpitations may be clearly and unequivocally distinguished from those which depend on organic disease of the heart or great vessels. This is an important diagnosis if always correct—which we fear it is not. "In nervous palpitation, says he, the pulsations of the heart are unequal, irregular, intermitting, sharp—the heart appears to bound—the extent of pulsation against the ribs is not greater than natural,—and the sound of the heart's action is not different *in kind* from that which is heard in health. These signs, though few in number, appear to me sufficient to distinguish nervous palpitation from all other irregular actions of the heart depending on organic diseases—while its occasional cessation, long duration, sudden disappearance and unexpected return—together with the absence of those symptoms peculiar to carditis, hydrothorax, &c. leave no doubt in the mind as to the nature of the affection."

The oppression, dyspnœa, œdema of the extremities, puffiness of the face, and blue tint of the lips, which sometimes accompany these nervous palpitations, depend, he observes on disturbance of the pulmonary circulation—and this disturbance is probably owing to want of rhythm or equilibrium of action between the right and left chambers of the heart. Dr. B. considers this kind of palpitation to be a serious disease—and

* Practitioners should recollect that the effects of digitalis, and of many other potent medicines, accumulate, as it were, in the system, and their explosion, when arrived at a certain point, is very dangerous, and not very unfrequently fatal. This is the case with colchicum, mercury, &c. For many excellent observations on digitalis, when carried too far, we refer to a paper of Dr. Maclean, in the second volume of the Medical and Physical Journal, page 113, *et seq.*

the more so, in proportion to its standing, and to its resistance to proper remedies. It may then, he thinks, determine aneurism, or enlargement of one or more cavities of the heart, and ultimately prove fatal.

Antispasmodics are the remedies on which practitioners have relied in this complaint; but he looks upon digitalis as almost a specific in nervous palpitation. He properly cautions the practitioner to watch the effects of the medicine, and immediately diminish the dose, or withdraw it entirely, whenever the pulse sinks below the healthy rhythm. He acknowledges that death in the third case, was probably owing to the digitalis completely paralysing the central organ of the circulation.—*N. Bibliotheque, Decembre*, 1825.

In the Journal Gen. de Medecine for Jan. 1826, we observe two interesting cases, related by M. Comte, in one of which there was strong reason to suspect organic disease of the heart, and in both of which, the digitalis was of essential service.

The first case was that of an unmarried lady, 35 years of age, who had been attacked with palpitation of the heart at the age of eleven years, in consequence of a fright, and which continued more or less violent for two years, and then the fits of palpitation became much less frequent, occurring only once a month or once in two months. This state continued till the age of 24, when the attacks were separated by still longer intervals—as three or four months. About this time she became affected with hæmoptysis for more than a year, and with the effect of removing the palpitation entirely till the age of 32 years. In 1823, they became very violent for nine months, returning several times every day, and at any time readily excited by the slightest moral emotion. They disappeared for three or four months—and, in September, 1824, returned, with alarming fits of syncope. On the 10th December, our author was called to this lady, who could not then move from her bed. The pulse was small and concentrated, with uneasy anxious feelings about the præcordial region, frequent palpitations, which could be seen and heard distinctly, face pale, no appetite, impossibility of sleeping, cold feet, menstruation irregular. Pills composed of camphor, nitre, opium, and digitalis were prescribed, every four hours, and produced a state of calm. The remedy was persevered in for some time, and the palpitations almost disappeared, except when excited by mental emotion. This ameliorated condition has continued up to the present time. But, it cannot be expected that a disorder (even of function) which has existed so many years, will cease all at once, under the best system of medicine or diet. The palpitations will doubtless return, with the causes that produced them; but there is fair reason for believing that the medicines prescribed above, have been beneficial in this case.

*Case.* The second case related by M. Comte, is detailed with great minuteness; and we shall be obliged to curtail it much of its fair proportions.

The son of the Baron de L———, an officer in the Guards, now nine years of age, had been very delicate and susceptible from his birth. One of his brothers had evidently an affection of the heart—his mother appeared to have the same organ in a very weak state—and another brother had died of disease of the heart, as verified by dissection. The youth whose case now occupies our attention, was pronounced, in the year 1822, by Leroux and Dupuytren, to have—"*a disease of the heart, consisting of an enlargement of that organ to a considerable extent, with inordinate pulsation throughout the whole of the left side, and dropsical effusion in both sides of the chest, in the abdomen, and in the cellular membrane generally.*"

After having tried some of the more common remedies, without effect, Leroux and Dupuytren ordered the powder of digitalis in doses of three or four grains daily, together with a liniment, in which the tincture of digitalis predominated. By these means the symptoms were very speedily dissipated, and the little invalid was restored to a considerable share of health, in the course of a month. But, soon afterwards, he began to experience palpitations, cough, and wandering pains in various parts of the body. For the three next years the youth was valetudinary, and unable to take the exercise or undergo the scholastic discipline of his equals in years.

On the 1st May, 1825, the young Baron was invited to a royal fête, and made rather too free with the sweet-meats and wine, for which he soon began to pay dearly. On the 5th of the same month our author saw him for the first time. The pulse was 130 in the minute—the pulsations of the heart were in correspondence, and shook the whole of the left side of the chest. On percussion, the chest sounded pretty well, except in the region of the heart, where there was a considerable projection of the ribs to the extent of six lines at least, and for a space of four inches in breadth. He had hard, dry cough;—the face was *pale*, and the countenance indicative of much suffering. Excepting the paleness of the face, the other symptoms led to the belief that there was an aneurismal affection of the left ventricle of the heart. The exception above-mentioned induced our author to conclude that the disease was a simple hypertrophy of the organ, carried to its highest degree, and which, though less dangerous than aneurismal dilatation of the left ventricle, was yet a formidable complaint, which must ultimately prove fatal.—Half a grain of the powder of digitalis was at first prescribed daily, to be daily increased, enjoying the most perfect quietude of body and mind, with the lowest possible scale of diet. 8th, The boy was better, and his nights more tranquil. He could lie down in bed—his pulse was reduced to 60 in the minute—the elevation of the ribs was much less prominent—the boy wished to go out into the garden to amuse himself, and had a return of appetite. Our author felt quite astonished and well he might, at this sudden change for the better; especially as the dose of digitalis had only yet amounted to three grains *per diem.* We forgot to mention, however, that hot pediluvia, with mustard, had been employed in the mean time, and that great importance was attributed by M. Comte to this remedy. We cannot well comprehend our author's pathology, as exemplified in the following passage.

"La prompte disparition de ces derniers symptomes, et la suite de cette observation donneront une juste idée de cette espèce d'hypertrophie du cœur, qui n'etait qu'un *boursoufflement spasmodique*, une exaltation accidentale de cet organe, plutot qu'un surcroit réel de volume, avec lesion organique."

*A Spasmodic Swelling or Puffiness of the Heart!* Really this reminds us of Oliver Hill's fifth Essay on the Circulation of the Blood, or rather *against* the circulation, shewing that the true cause of the motion of the heart, of the blood, and of the arteries, is—"*The spirits making a* FLASH *in the left ventricle, and a* PUFF, *which swells the heart at every pulsation*, and pervades and moves the blood."*

Be this as it may, the jeune garçon—we beg pardon, le jeune Baron, recovered rapidly, under the digitalis plan, and by the 15th his mother considered him "comme gueri d'une affection très-grave," of which her other son had died. But these halcyon days did not last long. On the 18th of the same month, the fond mother took the recovered son to the representation of JOCKO—by which he was amazingly amused and excited—but by which the mother averred he was by no means injured. On the 20th, however, our author found the young Baron as bad as ever, and, therefore, prescribed the digitalis and quietude, with low living, as before. The patient continued very ill till the 25th, when the symptoms were rather mitigated. Still there was great uneasiness and anxiety in the region of the heart, and excessive nervous irritability throughout the whole system. This state changed into one of great debility, with much tendency to somnolency and affection of the brain. Upon this supervened a high state of gastric irritation, if not inflammation, requiring leeches to the epigastrium. The projection of the chest, in the region of the heart, now almost disappeared, and the youth began to sit up a little and even attempt to walk, but without the power. At this time, viz. on the 29th, M. Laennec was requested to examine the boy's chest, and noted the following diagnosis :—"every part of the chest, *except the præcordial region*, renders the natural sound—in the latter it is doubtful, on account of the distention of the stomach by gas. Respiration natural throughout the chest—movements and noise of the heart nearly natural. *Diagnosis.*—Pericarditis ending by resolution." He prescribed antimony, digitalis, occasional leeching. He considered the palpitations and irregular action of the heart as owing to "a violent spasm settled on that organ." M. Comte did not, however, agree with M. Laennec as to the existence of pericarditis. Between the 1st and the 4th of June, the little patient suffered very much, without any evident cause, from oppression and fixed pain in the region of the heart. But our author, on seeing the boy pale, feeble, and dejected, with quick, soft pulse, considered the pain as spasmodic, and ordered semicupia, which had the desired effect. The digitalis was still continued. But we must bring this tremendous long case to a close. The boy sometimes rallied, and at others, was so near death, that the priest superseded the doctor, for the purpose of applying extreme unction.

* Hill on the Circulation, 8vo. London, 1700.

At one time too, another eminent physician was called in to consult with M. Comte, and pronounced the disease to be an aneurism of the heart, and that the young Baron had but a few days, perhaps only a few hours, to live. This prognosis was not verified; for the parents took the child down into Normandy, and returned several months afterwards with the invalid in a much better condition than when he left Paris. The action of the heart is still high, and we have much doubt whether the youth will not ultimately fall a victim to organic disease. This, however, is not the opinion of his physician, and he must be the best judge. The young Baron is safe for the present—and we have in him a good specimen of what the nervous system is capable of doing, and how dangerous it is to pronounce a *disorder* of the heart's action to be a disease of its structure.

